# A bio-inspired minimal model for non-stationary K-armed bandits

**DOI:** 10.1007/s00422-026-01037-5

**Published:** 2026-03-10

**Authors:** Krubeal Danieli, Mikkel Elle Lepperød

**Affiliations:** 1 Center for Integrative Neuroplasticity, FYSCELL, University of Oslo, Oslo, Norway; 2 Simula Research Laboratory, Oslo, Norway

**Keywords:** Decision-making, Reinforcement learning, Plasticity, Adaptation

## Abstract

While reinforcement learning algorithms have made significant progress in solving multi-armed bandit problems, they often lack biological plausibility in architecture and dynamics. Here, we propose a bio-inspired neural model based on interacting populations of rate neurons, drawing inspiration from the orbitofrontal cortex and anterior cingulate cortex. Our model reports robust performance across various stochastic bandit problems, matching the effectiveness of standard algorithms such as Thompson Sampling and UCB. Notably, the model exhibits adaptive behavior: employing greedy strategies in low-uncertainty situations while increasing exploratory behavior as uncertainty rises. Through evolutionary optimization, the model’s hyperparameters converged to values that align with the principles of synaptic mechanisms, particularly in terms of synapse-dependent neural activity and learning rate adaptation. These findings suggest that biologically-inspired computational architectures can achieve competitive performance while providing insights into neural mechanisms of decision-making under uncertainty.

## Introduction

The ability to make choices for long-term reward maximization is a fundamental aspect of cognition. The brain has evolved specialized and interconnected regions to implement this behavior within the constraints of biology.

An established framework for investigating decision-making is reinforcement learning and Bayesian Decision-Theory (BDT), which helped formalize some possible cognitive strategies in relation to reward (Sutton and Barto 1998). In this picture, the decision process may be based on past information as well as predictions about the future, whose results may affect the possibility of a positive or negative gain (Dayan and Daw 2008). Important lines of research in this regard have revolved around the underlying learning mechanisms, value estimation, and competition among conflicting options. Classical learning frameworks include Pavlovian conditioning, in which animals pair external cues with hard-wired responses (Allen and Madden 1985). Operant conditioning extends this by reshaping behavior through repeated experience (Baldassarre and Parisi 2000). A more computational view treats the brain as an agent or controller for making decisions (Dayan and Daw 2008). These approaches are typically classified as model-based or model-free, depending on whether behavior relies on an internal model of the environment or simply on a state–action mapping, with consequences for adaptability and computational efficiency (Sutton and Barto 1998).

Regarding brain implementation, it has been hypothesized that multiple controllers coexist, differing in their local circuitry, plasticity mechanisms, and position within a neuroanatomical hierarchy (Bach and Dayan 2017). Dopaminergic neurons in the ventral striatum of the midbrain have long been associated with model-free algorithms, such as temporal difference learning, given their involvement in action selection and the strong correlation with reward prediction errors (Suri 2002; Niv et al. 2005; Schultz 2016). Nevertheless, it has also been speculated that they contributed to model-based dynamics, in particular through the interplay with the orbito-frontal cortex (OFC), a region known for being involved in value representation (Frank and Claus 2006; Singh et al. 2010; Kennerley and Walton 2011; McDannald et al. 2012). Another area that has emerged for its role in option evaluation and selection is the anterior cingulate cortex (ACC), particularly during action selection, perceptual disambiguation, and multi-stability situations (Walton et al. 2007; Safavi and Dayan 2022). Indeed, there is consistent evidence that the interaction between OFC and ACC plays a crucial role in decision-making, integrating high-level emotional information sourced from the amygdala and the temporal and hippocampal regions (Rosenbloom et al. 2012; Luk and Wallis 2013; Balewski et al. 2023; Rolls 2023).

Well-studied ecological settings for decision making are foraging tasks, such as food search. In these problems, the agent is usually asked to choose between different options to maximize an expected reward. In nature, animals have been shown to exhibit different strategies depending on the context. *Matching behavior* is a well-known phenomenon in which the animal’s decision patterns are proportional to the reward probability of the available options. This behavior is believed to be the result of the trade-off between exploration and exploitation (Niv et al. 2002). In fact, this is a well known phenomenon in the reinforcement learning literature, in which an agent is faced with the dilemma of exploring new alternatives, potentially more rewarding, or exploiting known options, despite being possibly sup-optimal.

A popular formalization of these types of tasks is the *multi-armed bandit* problem (MAB) (Averbeck 2015). This setting is usually described in terms of a slot machine endowed with *K* distinct arms, also called levers. During a round, the agent selects one of the arms and collects a reward *R* according to an unknown probability of the specific reward for the chosen arm. The goal is simply to maximize the total reward after a given number of steps, which is achieved by effectively updating a selection policy after each round. This problem has been extensively studied in the context of reinforcement learning and is considered a fundamental building block for more complex tasks (Sutton and Barto 1998).

The multi-armed bandit problem comes in several variants, with the simplest featuring a stationary reward distribution. An important performance measure in these tasks is *regret*, usually defined as the distance between the selected choice and the theoretically optimal one. Researchers have proposed numerous algorithms to address this problem, each with distinct theoretical guarantees.

Thompson sampling (TS) is a widely adopted approach rooted in Bayesian optimization. It maintains a posterior distribution over action reward probabilities and selects actions by sampling from these distributions. Thompson sampling has demonstrated near-optimal regret bounds in stochastic settings (Agrawal and Goyal 2012; Kaufmann and Korda 2012). In contrast, the Upper Confidence Bound (UCB) algorithm uses an optimistic principle for exploration. It maintains an estimate of the reward for each option by a confidence interval. Action selection relies on the upper bound of this interval, encouraging exploration of less-visited options by assigning them higher uncertainty. UCB has been shown to achieve logarithmic regret in stochastic bandits (Auer et al. 2002). Another effective baseline is the $\epsilon$-Greedy strategy. At each decision step, a random action with probability $\epsilon$ and the best known action with probability $1 - \epsilon$ (exploitation) is selected. Although not as theoretically optimal as Thompson Sampling or UCB, $\epsilon$-Greedy is simple to implement and often effective in practice. Extensions such as VDBE adapt $\epsilon$ dynamically based on the variance of the value function, providing better control over exploration (Gittins 1979; Ban et al. 2021; Tokic 2010; Tokic and Palm 2011).

However, these traditional algorithms, despite their effectiveness, lack biological plausibility – they neither resemble neural circuits nor follow synaptic plasticity dynamics. For example, they do not rely on a network-like architecture with interconnected units, as seen in the brain. Additionally, their action selection process is typically instantaneous, whereas decision making in the brain occurs over time, often requiring the activity of a neural circuit to evolve and stabilize before converging on a final selection. Their learning mechanisms also differ fundamentally. They typically involve explicit updates to statistical parameters (e.g., reward estimates or exploration rates) based on observed outcomes. In contrast, biological learning relies on local plasticity rules, where synaptic changes depend on the activity of connected neurons, modulating how input is integrated and how output signals are generated. Although not the primary driver, these limitations align with a growing interest in machine learning towards bioinspired algorithms, such as neural networks and predictive coding (Lee et al. 2022; Spratling 2017), which offer several advantages.

In fact, some of these biological cognitive systems can achieve state-of-the-art performance in various domains, including the challenging *machine-challenging tasks* (MCTs), set of problems that are difficult for machines but relatively easy for humans (Schmidgall et al. 2024; Hassabis et al. 2017; Lee et al. 2022). In addition, bioinspired models improve algorithmic interpretability by clarifying the functional relationships between internal components. When applied to tasks with existing experimental data, these models can generate new insights into the brain and suggest new research directions (Liu and Gan 2023). Although other approaches such as Bayesian learning can match human behavior (Behrens et al. 2007) and brain data (Tomov et al. 2020), they fail to capture the underlying neural mechanism.

In this work, we aim to enhance the biological plausibility of models used in multi-armed bandit tasks by introducing a novel, minimal decision-making architecture called Neural Selection Agreement (NSA) model. This model comprises two interacting rate-based neuronal populations connected by plastic synapses, uses a biologically inspired plasticity rule, and forms decisions based on the agreement of the two populations on the next option.

The model’s plasticity mechanism is non-Hebbian and depends on the magnitude of inter-population synaptic weights. This formulation aligns with synapse-type specific plasticity (STSP), a biologically supported mechanism linking learning dynamics to synaptic resource availability, current state, and morphological properties (Larsen and Jesper Sjöström 2015; Blackman et al. 2013; Bartol et al. 2015; Ariel et al. 2012). Learning rates are also adaptive, in line with observations in human experiments (Behrens et al. 2007).

Similar forms of plasticity have been employed in prior work on spiking neural networks and models of synaptic metaplasticity (Inglis et al. 2021; Iigaya et al. 2016). Despite its simplicity, our model performs comparably with standard algorithms such as Thompson Sampling, $\epsilon$-Greedy and Upper Confidence Bound, while offering a more neurobiologically grounded account of decision-making. Other studies have also proposed solutions to bridge reinforcement learning and neural mechanisms. For example, Starkweather et al. (2018) proposed temporal difference algorithms of the belief state (Babayan et al. 2018) that abstract dopaminergic signaling and medial prefrontal projections. These models highlight the role of hidden-state inference during probabilistic tasks, although they assume fixed reward distributions and do not incorporate explicit synaptic plasticity.

In Khorsand and Soltani (2017), metaplasticity mechanisms were explored in relation to the probability estimation of binary sequences, uncovering informative patterns of functional synaptic states. However, the environment varied along a single stimulus dimension. Similarly, Farashahi et al. (2017) applied a metaplasticity model to a probabilistic reversal learning task, effectively a stationary two-armed bandit, revealing the emergence of option-specific learning dynamics. A notable exception is Iigaya (2016), which addressed a non-stationary two-arm bandit using a synaptic cascade model (Fusi et al. 2005) equipped with a surprise detection mechanism to track changes in reward probability.

In contrast to previous work, our study addresses more challenging, high-dimensional nonstationary reward environments, including up to 1,000 arms with independently drifting reward probabilities. We specifically focus on stochastic bandit problems with *concept drift*, where reward distributions evolve over time, either gradually or through abrupt changes, thus requiring flexible and adaptive decision-making strategies (Garivier and Moulines 2008; Besbes et al. 2014; Cavenaghi et al. 2021). Despite its simplicity, the proposed model performs competitively with standard algorithms such as Thompson Sampling, $\epsilon$-Greedy, and Upper Confidence Bound, while offering a more biologically grounded perspective on decision-making mechanisms.

In general, our work aims to bridge adaptive decision-making under uncertainty with principles from computational neuroscience. By proposing a biologically plausible mechanism for choice behavior in dynamic environments, we contribute a framework that may inform both the development of adaptive artificial systems and the interpretation of neural processes underlying flexible behavior.

The remainder of this paper first describes our model design and learning, then presents experimental results and comparative analyzes with established algorithms, and lastly discusses the findings’ broader implications and potential future directions.

## Methods

The following section is organized as follows. First, we introduce a formalization of the general problem setting, together with the variants considered in this work. Then we outline the architecture of our model and how it can be mapped to neurobiology. Finally, we describe the learning procedure and showcase its dynamics in a simple example.

### Binomial MAB problem

The standard formulation of the task is structured as a set of *K* arms (or levers) $\mathcal {A}_{K}=\{a_{1}\ldots a_{K}\}$, with associated reward probabilities $\textbf{p}=\{p_{1}, \ldots p_{K}\}$. At each iteration, the agent pulls an arm and collects a possible reward drawn as a Bernoulli variable $R\sim \mathcal {B}(p_{k})$. The agent’s objective is to maximize the total reward $\sum ^{T}_{t} R_{t}$, after a certain number of rounds *T*, also called the horizon. Importantly, the agent is unaware of the true probability of reward and therefore must make its decisions following a certain policy, denoted $\pi$. In the reinforcement learning literature, the policy is often defined as a distribution over actions, here the arms $\mathcal {A}_{K}$, given the current state at time *t*. In the bandit problem, the state can be taken to correspond to the history $h_{t}$ of past actions and rewards in the period $\{0\ldots t\}$, and the policy as a function that returns a selected arm $\pi (h_{t})=a_{t}$ (Qi et al. 2023).

Given the inherent stochasticity of the feedback from the environment, the policy is affected by the so-called exploration-exploitation trade-off, which here is phrased as the contrast between the option of the arm with the estimated highest expected reward versus the option to explore other arms, so as to gather more information. A common approach is the $\epsilon$-Greedy policy, where the choice to explore is selected with probability $\epsilon$. Moreover, it is often preferable to have more exploratory behavior early during the training, with the intent to have a good sample size for the empirical reward distribution, which can be later exploited for maximizing reward.

Another important concept in multi-armed bandit problems is *regret*. Intuitively, it quantifies the loss of reward due to following a certain policy, and it is determined by the difference between the collected reward and the theoretical optimum, obtained by choosing the best arm at each round. Formally, given a function $r(\pi )$ that returns the expected reward while following policy $\pi$, regret $\rho$ over a horizon *T* can be formulated as

1 $$\begin{aligned} \rho = \frac{1}{T}\sum _{t=1}^{T} \left( p^{*}_{t} - r(\pi (h_{t}))\right) \end{aligned}$$

where $p^{*}_{t}$ is the expected reward of the optimal arm at time *t*, which corresponds to its probability since it is a Bernoulli distribution.

The goal of the agent is to minimize the regret and thus maximize the total reward.

### Neural selection agreement model (NSA)

The model is constructed as a rate network composed of two neuronal populations, U and V. The first (U) represents the traces of the *K* available options (that is, the bandits), while the second, V, encodes their values according to the current policy.

**Fig. 1:**
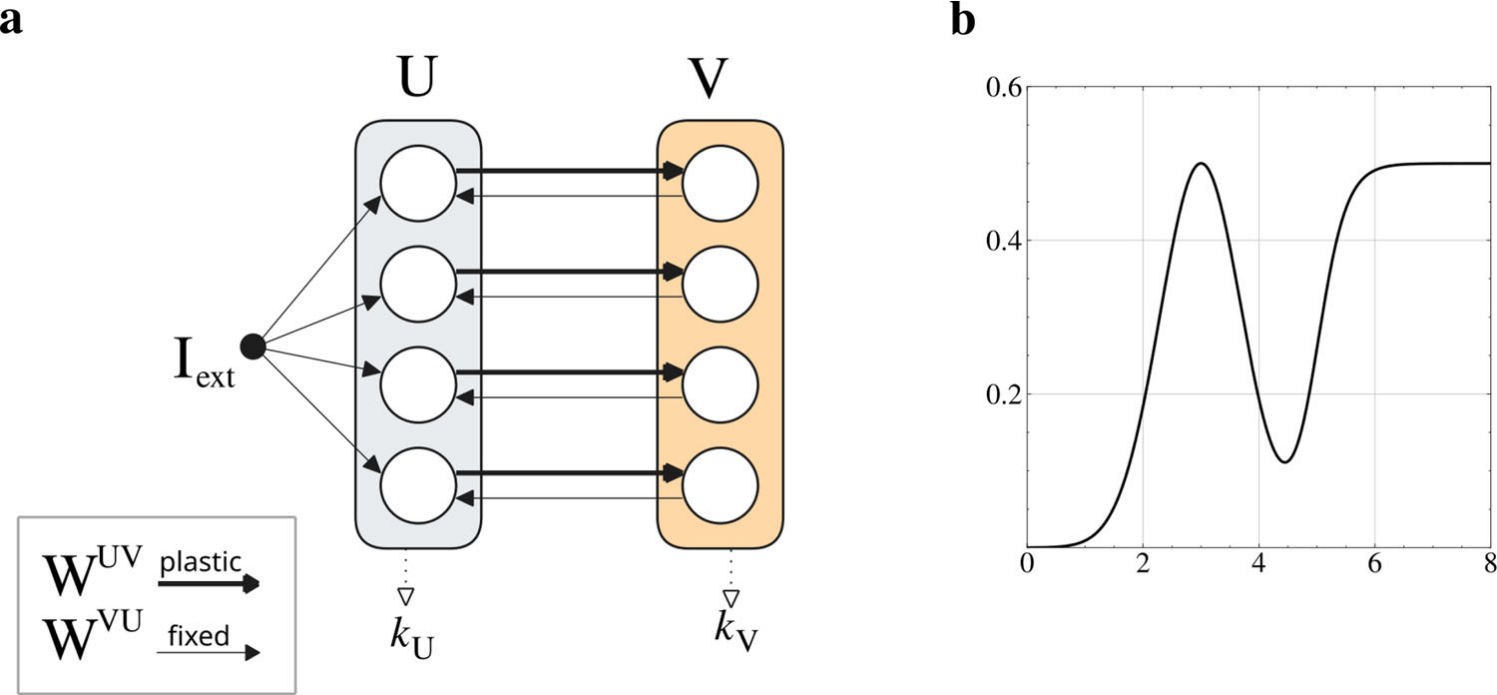
Model architecture - **a**: the model is composed of a layer *U* (grey), receiving a feedforward input $I_{\text {ext}}$, a layer *V* (orange), and connections ${\textbf {W}}^{UV}$ and ${\textbf {W}}^{VU}$. Additionally, two indices $k_{U}, k_{V}$ are extracted from the layers and correspond to the selection made by the two populations as $k_{U}=\text {argmax}_{k} \{{\textbf {u}}\}$, $k_{V}=\text {argmax}_{k} \{{\textbf {v}}\}$. - **b**: the function used to filter the weights ($\Phi _{v}$) and the learning rate ($\Phi _{\eta }$). It is defined as a weighted sum of a generalized sigmoid and a Gaussian function, both of which have additional parameters controlling their offset and slope steepness (or width). For more details about its implementation, see the Appendix 5.2.

In the model, the first layer represents the available options, while the learned connections to the second layer encode their values based on recent reward history. In Fig. 1a a visualization of the architecture is presented. A key simplification is the lumping of option representations into single neurons. Although this choice abstracts the more distributed encoding found in actual brain networks, it allows for a more tractable model design (Martin 2007).

More formally, the model is defined by a set of coupled ordinary differential equations (ODEs). The first equation describes the evolution of neural activity ${\textbf {u}}$ in population U, while the second governs the activity ${\textbf {v}}$ in population V, each evolving with its respective time constant $\tau$.

2 $$\begin{aligned} \begin{aligned} \tau _{u} \dot{{\textbf {u}}}&= -{\textbf {u}} + {\textbf {W}}^{VU}\phi _{v}({\textbf {v}}) + {\textbf {I}}_{\text {ext}} \\ \tau _{v} \dot{{\textbf {v}}}&= -{\textbf {v}} + \widetilde{{\textbf {W}}}^{UV}\phi _{u}({\textbf {u}}) \end{aligned} \end{aligned}$$

The external input ${\textbf {I}}_{\text {ext}}$ is a constant input that is used to set the initial conditions of neural activity ${\textbf {u}}$. Notably, it does not deliver any practical information, given the lack of ambiguity of the task, and should therefore be treated more as an attention-related stimulation to prompt processing (Yantis 2008). The activation functions $\phi _{v},\phi _{u}$ are applied to population *v* and *u* respectively, and represent two distinct neural response functions tailored to each population vector. They have been chosen to be a step function with threshold $\theta _{v},\theta _{u}$ applied to a generalized sigmoid with gain $g_{v},g_{u}$ and offset $s_{v},s_{u}$.

Importantly, the two layers are not fully connected and the matrices are diagonal. Moreover, the weight matrix ${\textbf {W}}^{VU}$ is a diagonal matrix made of 1s and is fixed, while $\widetilde{{\textbf {W}}}^{UV}$ is a function of the actual weights $\Phi _{v}({\textbf {W}}^{UV})$ and represents the contribution of the active options ${\textbf {u}}$ to the value representation ${\textbf {v}}$, so it is called *the option value function*. The matrix ${\textbf {W}}^{UV}$ is initialized to all zeros and is the only plastic parameter of the model during the task. In terms of other parameters, they were optimized offline with the goal of maximizing the average total reward over multiple runs and across testing environments. In particular, given the non-differentiability of the model with respect to the fitness function, we relied on an evolutionary search using the Covariance-Matrix Adaptation algorithm (CMA-ES) (Igel et al. 2007). The function $\Phi _{v}$ is defined to be a combination of a Gaussian and a sigmoid, and its parameters were also optimized offline. Figure 1b illustrates an example. The motivation behind this choice is to offload the responsibility of converging to a shape useful for solving the task to evolution.

The main difference between the two functions is the presence (sigmoid) or absence (Gaussian) of a saturation limit on either side of their maximum, with variable offset (threshold) and steepness (gain). Our intention was to explore the effect on performance of the integration and competition of these traits. Furthermore, these features are consistent with some characteristics of biological and artificial neurons, such as firing rate functions being smoothly bounded and receptive fields being defined in a range (Butts and Goldman 2006; Miller and Cannon 2019; Ocker and Buice 2020; Apicella et al. 2021).

Nevertheless, in addition to these reasons, another basis function could have been chosen with possibly not dissimilar results.

#### Option selection

The decision-making process within a single round is structured in two distinct phases. Initially, the model receives a constant external input targeting all neurons in the population U equally. During this phase, ${\textbf {I}}_{\text {ext}}$ works as an equilibrium value while reciprocal interactions with population V push ${\textbf {u}}$ to different values, depending on the current policy encoded in $\widetilde{{\textbf {W}}}^{UV}$. Importantly, the weights ${\textbf {W}}^{UV}$ are initialized to zero, and thus the input from *U* to *V* is uniform. This approach ensures the absence of biases towards any arm by having all weights equal, and corresponds to a completely untrained network. The neural dynamics 2.2 is simulated by using a first-order explicit Euler integration method, using a time discretization of 1 (in milliseconds). After a fixed time duration $\text {dur}_{\text {pre}}$s, the second phase begins. Here, the external input is removed and the model is left to evolve autonomously, and since there are no recurrent connections in either population, the dynamics are entirely driven by their coupling. A selection *k* is sampled after another fixed duration $\text {dur}_{\text {post}}$, and is defined according to the following rule:

$$\begin{aligned} k = \left\{ \begin{array}{ll} \text {argmax}_{k}\{{\textbf {v}}\} & \text {\textit{if}}\; \text {argmax}_{k} \{{\textbf {v}}\} = \text {argmax}_{k} \{{\textbf {u}}\} \\ \text {random}(K) & \text {\textit{otherwise}} \end{array} \right. \end{aligned}$$

This rule presents a way to express the exploration-exploitation trade-off through the possible agreement of the two populations, under the influence of current weight values $\widetilde{{\textbf {W}}}^{UV}$.

In subsect. 2.2.1, the pseudo-code for the algorithm behind the selection process is reported below, which is applied during each round *t*. The two duration hyperparameters $\text {dur}_{\text {pre}}$ and $\text {dur}_{\text {post}}$ evolve together with the others within a range of (400, 3000)ms. Although this range has been defined based on empirical motivations and closely depends on the settings of our model, at least for cases with few options it aligns data from experiments measuring reaction times, typically on the order of 300–1500 ms (Churchland et al. 2008; Drugowitsch et al. 2014).

**Algorithm 1:**
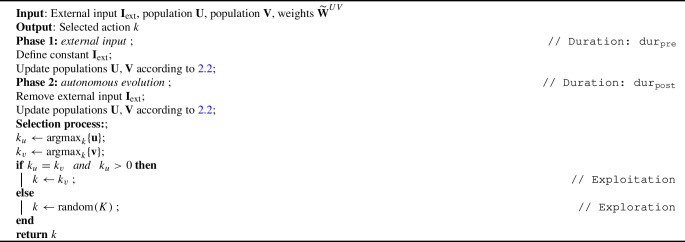
Two-phases option selection process

In the computation of $\text {argmax}$, in the event of a tie between options, the first result is taken. In fact, empirically, no difference has been observed from random sampling, and intuitively, in the long term, the subset of rewarding options will shrink with learning. According to the values of the policy parameters, the behavior of the model displays periods of exploration followed by steady exploitation, which can be reverted in the case of a change in the environment’s reward distribution.

The structure of this option selection process draws inspiration from the interaction between the values estimation, feedback integration, and option representation produced by the orbito-frontal cortex (OFC) and the anterior cingulate cortex (ACC) (Kennerley and Walton 2011; Luk and Wallis 2013; Rolls 2023). In particular, consensus-like dynamics have been proposed for selecting actions, evaluated in a parallel and distributed fashion, and undergoing competition (Cisek and Kalaska 2010; Cisek 2012; Suzuki et al. 2015). Another relevant perspective is that of bump attractor models for perceptual decision-making, usually defined as recurrent networks trained to drift within their activity landscape to solve multi-choice tasks (You and Wang 2013; Carroll et al. 2014; Esnaola-Acebes et al. 2021).

### Learning

Given a selected option *k*, the environment (set of bandits) samples and returns a reward $R=1$ with probability $p_{k}$. Then, the weights ${\textbf {W}}^{UV}$ for the neuron corresponding to option *k* are updated according to the following plasticity rule.

3 $$\begin{aligned} \Delta {\textbf {W}}^{UV}_{k} = \tilde{\eta }_{k} \left( R\cdot w^{+}- {\textbf {W}}^{UV}_{k}\right) \end{aligned}$$

where $w^{+}$ is a constant maximum synaptic weight, while $\tilde{\eta }_{k}$ is the learning rate for the option *k* determined by a function $\Phi _{\eta }$ of the current weights ${\textbf {W}}^{UV}_{k}$, known as the *learning rate function*.

The shape of $\Phi _{\eta }$ is again a Gaussian-sigmoid but with different parameters, giving evolution the opportunity to combine the two characteristic traits of smooth saturation and bell-shaped tuning. In terms of neural dynamics, the advantage is to introduce adaptation in the learning of the weights, implemented as a more considerate scaling of the update magnitude. Computationally, this allows taking into account different learning phases for a given neuron, which represents an option, and an approach similar to the adaptive optimizers employed in training deep networks (Wen and Zhou 2024). In addition, proportional updates and synaptic scaling have been observed in biological synapses, including plasticity mechanisms, such as shaping the synaptic update kernel, or homeostatic constraints, for instance, limits on the speed of chemical exchange in the synaptic cleft (Citri and Malenka 2008; Larsen and Jesper Sjöström 2015; Kennedy 2016; Samavat et al. 2024).

## Experiments

The NSA model was tested in a series of benchmark environments, each with a different number of arms and reward distributions. Performance was compared with the following algorithms: Random Baseline, Upper Confidence Bound (UCB), Thompson Sampling, and $\epsilon$-Greedy. The first two of these were implemented without any pre-defined hyperparameters, such that they automatically balanced exploration and exploitation through experience. However, $\epsilon$-Greedy required tuning of its hyperparameter $\epsilon$, for which we defined a rule based on the number of options of the form $0.4 \cdot \log _{2}(0.6K)$, whose specific values were fitted empirically (see Appendix 5.3.3).

### Game variants

**Fig. 2:**
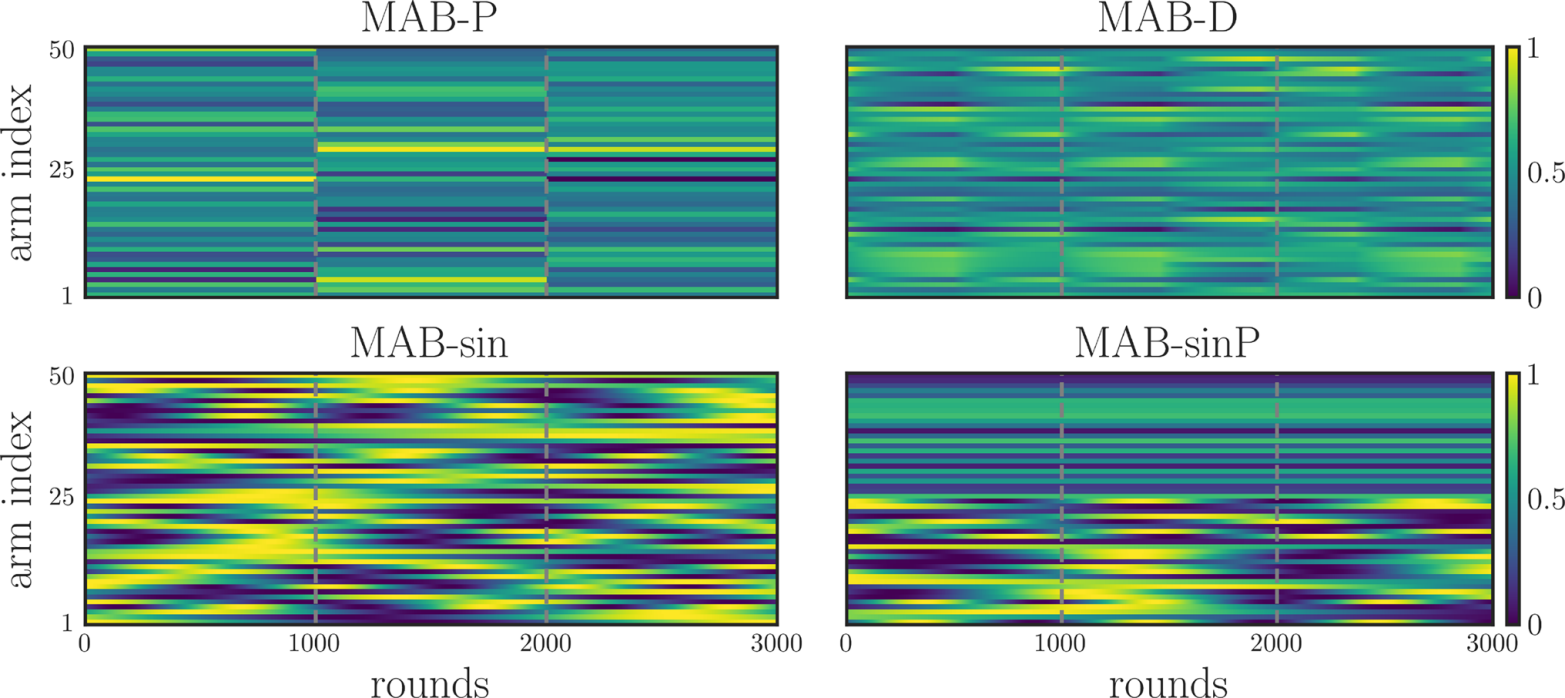
Reward distributions for the four MAB variants – the reward distributions for each variant are illustrated: piecewise stationary (MAB-P), piecewise stationary with drift (MAB-D), sinusoidal shift (MAB-$\sin$), and partial sinusoidal shift (MAB-$\sin$P). Arms are organized in rows; grey dashed lines demarcate trials (three), which are blocks of rounds represented by columns. Only the arm reward probabilities are shown

Our goal is to investigate the performance of the agent in a non-stationary environment, meaning that its underlying reward distribution changes over time.^1^ We choose this setting as it resembles an environment in which an animal forages for food (reward) distributed across a set of fixed locations, whose occurrence probabilities can change over time. A *round* (or horizon) is defined as a single action–reward event; a *trial* is instead a block of rounds. For testing, four slightly different MAB variants were used, obtained by introducing different types of non-stationarity: piecewise constant, uniformly changing, sinusoidally changing, and sinusoidally changing with piecewise constant arms. The reason for these choices is to test the performance of the model at different speeds and uniformities in the distribution changes. Figure 2 visually illustrates their specificities.

**Piecewise stationary distribution** [MAB-P]

Within a trial, the reward distribution is stationary and is drawn from a normal distribution $\textbf{p} \sim \mathcal {N}(0.5, 0.2)^{K}$, clipped to (0, 1). At the end of each trial *i*, a new distribution $\textbf{p}_{i} \rightarrow \textbf{p}_{i+1}$ is drawn (Qi et al. 2023).

**Piecewise stationary distribution with drift** [MAB-D]

Initially, the reward distribution $\textbf{p}$ is sampled from a normal $\textbf{p}=\mathcal {N}(0.5, 0.2)^{K}$. Then, it changes gradually over the rounds, tracked over time *t*, such that its values evolve toward a target distribution $\textbf{q}_{i}$ according to $\tau _{p} \dot{\textbf{p}}_{t} = \textbf{q}_{i} - \textbf{p}_{t}$. Here, $\dot{\textbf{p}}$ is the time derivative of the distribution, and $\tau _{p}$ is its time constant. Once the distance is below a threshold $\delta$ as $\vert \textbf{q}_{i} - \textbf{p}_{t}\vert < \delta$, the target distribution is changed to a new one $\textbf{q}_{i}\rightarrow \textbf{q}_{i+1}$. In this variant, there are no proper trials, but the target distribution continues to change until a maximum number of rounds is reached.

**Sinusoidal distribution shift** [MAB-$\sin$]

The reward distribution changes over rounds, with the probability of each arm following a sine wave with a specific frequency $f_{k}$, phase $\lambda _{k}$ and amplitude 1. At any given time *t*, the distribution is $\textbf{p}_{t}=\{\sin (2\pi f_{k} t+\lambda _{k})\text { for }k=1\ldots K\}$.

**Partial sinusoidal distribution shift** [MAB-$\sin$P]

Identical to the sinusoidal distribution shift, half of the arms change sinusoidally, while the other half is always kept at a constant value. The distribution is not normalized.

### Evolutionary search

**Fig. 3:**
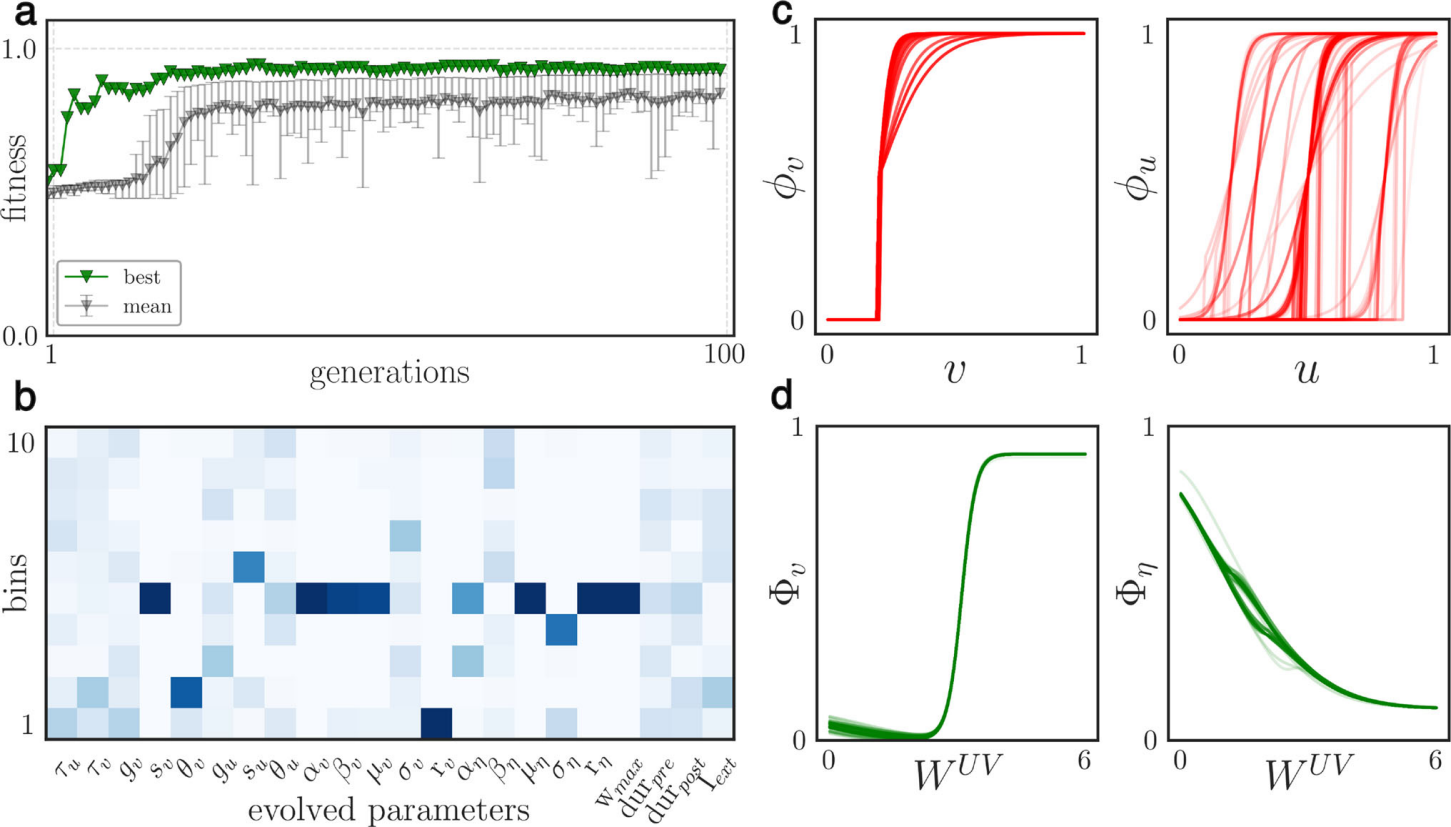
Evolution results - **a**: top fitness, mean and standard deviation (as 16–84 percentile) of the population over generations. - **b**: heatmap of the evolved parameters (rows) as histogram bins (y-axis) calculated from the 50 percentile of the population of the last generation; higher density is in dark blue - **c, d**: neural response functions (**c**) and Gaussian-sigmoid (**d**) of the top-half of the population, the color intensity is proportional to the fitness.

The optimization of the hyper-parameters was performed using the Covariance Matrix Adaptation evolutionary strategy algorithm (CMA-ES). The search was carried out with a population of 256 individuals for 80 generations. Each individual was endowed with a genome corresponding to a vector of 22 parameters of the model. The fitness function of the evolution was defined as the average reward obtained by an individual over 3 different non-stationary bandit environments, each for $K=\{40, 200\}$, and all averaged over 2 iterations. The results are summarized below in Fig. 3.

The evolution progress, plot 3a, showed a steady improvement over generations, with the fitness hitting a plateau once the total reward could not get any closer to the optimum $\approx 1$ due to randomness and the finite horizon.

The evolved parameters (constituting a *genome*) are displayed as a frequency heatmap in 3b, which was obtained from individuals whose fitness was equal to or above the median. The parameters that most strongly influenced the results were those directly associated with neuronal activity and learning. For each, the sensitivity of each value with respect to performance is inversely proportional to the variance its distribution over the population (related to the intensity of the heatmap color in 3b). The intuition is that the higher the variability, the smaller is the number of top-scoring genomes sharing a similar value.

Regarding the neural response functions shown in 3c, both populations evolved to have a similar shape, a sharp sigmoid with a clear threshold, with population *U* having a more variable distribution. The form is characterized by not allowing for a fine-grained linear response but rather a high-pass filter, with activity occurring only after strong excitation. This firing behavior is reminiscent of coincidence detector neurons, which are sometimes referred to as class III neurons with respect to their f-I curve (Ratté et al. 2013).

The two Gaussian-sigmoid functions $\Phi _{v}, \Phi _{\eta }$, which are, respectively, the option value and learning rate functions, are instead shown in 3d. On the one hand, the learning rate function $\Phi _{\eta }$ is characterized by a decreasing curve, associated with the dominance of the right-side of the Gaussian, and a completely ignored sigmoid component. A possible interpretation for this shape is to ensure a high learning speed when the value options are more uncertain (weak weights) and low otherwise; thus preventing overshooting and oscillations in the weight updates.

On the other hand, the option value function $\Phi _{v}$ instead follows a steep sigmoid curve. This is consistent with the idea that the input of population *U* to population *V* is maximally weighted for high option values (strong synapses), whereas for weaker estimates the contributions are low or close to zero, allowing for more exploration. Interestingly, a common feature seemed to be a slight concavity after zero, a slim influence of the Gaussian component, which might be interpreted as a sort of test for newly formed synapses. However, the size of this effect is not large.

### Environment variants and number of arms

The NSA model has been tested and compared with the other algorithms: Thompson Sampling (TS), UCB, and $\epsilon$-Greedy. The benchmarks were the four different variants of the MAB problem listed above 3.1, with a variable number of arms ranging from 5 to 1000. The results are reported in Table 1. Overall, our NSA model displayed competitive performance in all environments, often in the range or even better than the other algorithms. Interestingly, a large number of arms (*K*) did not present a significant challenge to our model, especially compared to the others. However, this result may be affected in part by randomness in the assignment of arm probabilities and the statistical increase of the count of high-reward arms as more are present. Considered the non-stationarity of the reward distribution, recalibrate to new distributions is still not a trivial task to accomplish. The NSA model encountered more difficulties, in comparison, in the second environment (piecewise stationary distribution with drift, MAB-D), making a positive difference only with the increase in arm number. A possible explanation is that MAB-D has a proportionally lower density of high-reward options at any given time, less compatible with the exploitative strategy emerging in the model. This is not the case for MAB-P, whose best arms have fewer but long lasting, and the sinusoidal MAB-$\text {sin}$ and MAB-$\text {sin}$P, which have more highly rewarding arms although changing frequently.

**Table 1:** Table of performance – from top to bottom: results for MAB-P, MAB-D, MAB-$\sin$, and MAB-$\sin$P, for different numbers of arms *K*.

	K	5	10	50	100	200	1000
MAB-P	TS	0.06	$\mathbf {0.06}$*	0.10	0.11	0.13***	0.29***
	$\epsilon$-Greedy	0.10	0.11	0.19***	0.22***	0.23***	0.25***
	UCB1	$\mathbf {0.05}$*	0.07	0.25***	0.36***	0.41***	0.49***
	NSA	0.10	0.11	0.11	0.11	$\mathbf {0.10}$	$\mathbf {0.10}$
MAB-D	TS	$\mathbf {0.09}$***	$\mathbf {0.08}$***	$\mathbf {0.17}$***	$\mathbf {0.23}$***	0.30	0.41***
	$\epsilon$-Greedy	$\mathbf {0.11}$**	$\mathbf {0.10}$***	$\mathbf {0.19}$***	$\mathbf {0.23}$***	$\mathbf {0.27}$***	0.33***
	UCB1	$\mathbf {0.10}$***	$\mathbf {0.11}$***	0.29***	0.38***	0.44***	0.49***
	NSA	0.15	0.17	0.25	0.29	0.31	$\mathbf {0.30}$
MAB-$\sin$	TS	0.22***	0.17***	0.10***	0.10***	0.10***	0.26***
	$\epsilon$-Greedy	0.29***	0.24***	0.18***	0.18***	0.17***	0.22***
	UCB1	0.11	0.09***	0.20***	0.26***	0.34***	0.51***
	NSA	0.08	$\mathbf {0.05}$	$\mathbf {0.06}$	$\mathbf {0.06}$	$\mathbf {0.06}$	$\mathbf {0.06}$
MAB-$\sin$P	TS	0.16***	0.20***	0.14***	0.12***	0.14***	0.28***
	$\epsilon$-Greedy	0.20***	0.27***	0.23***	0.22***	0.23***	0.27***
	UCB1	0.09	0.11	0.23***	0.32***	0.41***	0.56***
	NSA	0.06	0.09	$\mathbf {0.07}$	$\mathbf {0.07}$	$\mathbf {0.08}$	$\mathbf {0.08}$

Each cell reports the average regret, while stars represent the level of statistical significance of the difference with respect to the NSA model, calculated using Welch’s independent two-sample t-test with Bonferroni correction for multiple comparisons. Values in bold indicate either the models that perform meaningfully better than the NSA, or cases in which the NSA outperforms all others. The results were obtained over two trials of 2000 rounds each and averaged over 64 simulations

### Analysis of dynamics and robustness

#### Entropy analysis

**Fig. 4:**
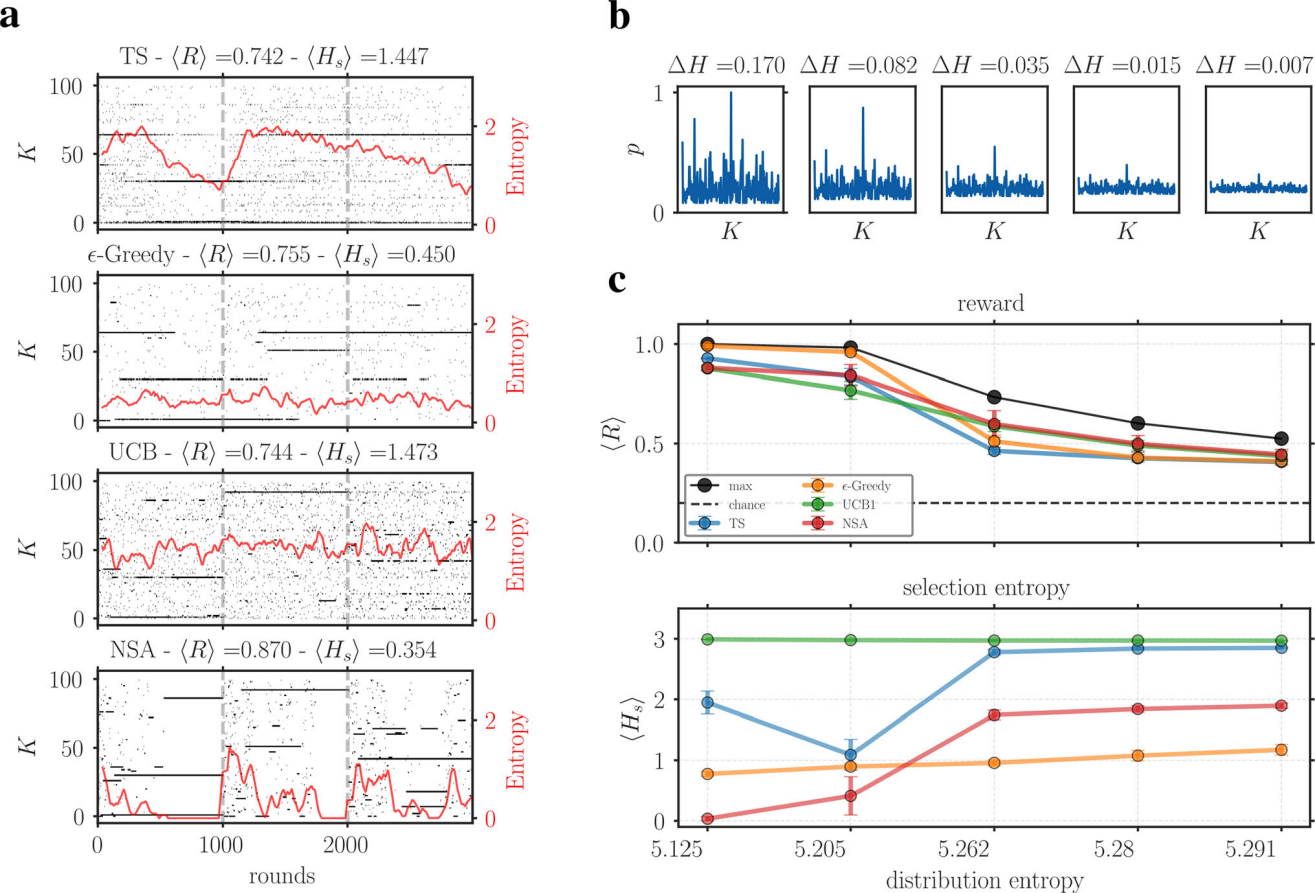
Decision-making dynamics for different models - **a**: each plot displays the results from one model. Raster plots (black dots) show the arms selected at each round. Red lines indicate entropy, calculated from the distribution of selections over the preceding 20 rounds, smoothed with a 30-steps moving average. Titles report total reward and average entropy. - **b**: examples of arm probability distributions ($K=200$) with increasing entropy. Subplot titles report the difference $\Delta H$ with respect to a reference uniform distribution with the same mean. - **c**: top sub-plot displays the average reward $\langle R\rangle$ for one trial obtained by each model for increasing levels of distribution entropy in the reward distribution. Bottom sub-plot shows the average entropy of the selections $\langle H_{s}\rangle$ for the same trial. The simulation used a stationary piecewise environment (MAB-P) with $K=200$, 2000 rounds, and averaged over 40 repetitions.

To better understand the qualitative differences between the models, we analyzed the decision dynamics by tracking the selected arms in a piecewise stationary distribution environment with $K=200$. Simulations were run for 2 trials with 2000 rounds each and averaged over 40 iterations. To quantify the variability of the decision policy at a given time, we computed the entropy of the probability distribution *p* of the chosen arms, calculated over a sliding window of 20 round, $H=-\sum ^{K}_{i} p_{i}\log (p_{i})$. The unit of entropy is in nats, and it ranges from 0 (no uncertainty) to $\log _{e}(K)$ (maximum uncertainty). In Fig. 4a, the raster plot of the selected arms is plotted for each model together with its level of entropy. The distribution of the probability of reward in the arms has an average of $H=2.02$.

As expected, the shape of the entropy curve expresses the inherent strategy adopted by each model. In particular, the UCB algorithm showed the highest variability, marked by persistent exploratory behavior throughout the trials despite converging to reward options. Thompson Sampling was able to reach most solutions, although it had difficulty adapting to new reward distributions that led to high entropy levels. $\epsilon$-Greedy also showed a good performance quite reliably, with the greedy strategy ensuring low entropy for most rounds. Similar behavior was observed for NSA, which was able to reach the optimal policy and maintain it over time, with the entropy peaking mostly at the beginning of the trials and being, on average, the lowest among all models. Indeed, the dynamics of NSA makes it particularly suited for the task of non-stationary MAB, as it is able to quickly adapt to new reward distributions and firmly maintain a greedy policy.

Then, we sought to investigate the robustness of NSA by targeting the capacity to endure increasing levels of entropy in the reward distribution, defined as $-\sum ^{N}_{i} p_{i} \log (p_{i})$ and calculated in nats. Figure 4b presents an example of arm distributions with increasing entropy, highlighting the blending, in terms of reward probability, of the best arm with the others, making the outlook more homogeneous and uniform. For more details on the implementation, see the Appendix 5.6. The investigation used a simulation carried out in a piecewise stationary environment with $K=200$, the results are plotted in Fig. 4c.

The analysis tracked performance, as cumulative reward, as well as selection entropy $H_{s}$, defined as the entropy of the distribution of selected options within a sliding window of rounds 20. In the upper sub-plot of 4c, the average reward obtained by each model is shown against the entropy of the reward distribution. The results reported that all models were capable of keeping up with increasing uncertainty and that the NSA model was not markedly different from the others. A noticeable performance was that of $\epsilon$-Greedy, which weakened only when the entropy increased significantly. The high levels of entropy of the last arm distribution made the choice of arms less important, meaning that the optimal selection policy was getting closer to chance levels (the dashed line), reflected by the similar performance across models.

Another perspective on this analysis was given by the bottom sub-plot, which showed the average entropy over the trials. Overall, there was an unsuprising trend of increasing selection entropy with the entropy of the reward distribution. However, striking is the exception of Epsilon-Greedy, which still maintained a constant level throughout; it is $\epsilon$ parameter was, however, only calibrated with respect to the number of arms and was therefore kept constant within the simulation. UCB displayed the highest average values, while Thompson Sampling followed with some delay, behavior also observable on the left in plot 4a. On the other hand, NSA displays a more abrupt change, going from a state of very low to high variability, a sign of more pronounced exploratory behavior as the best arms become harder to spot and hold.

## Discussion

The ability to make decisions under uncertainty is a fundamental aspect of cognition. A well-established framework for studying this capacity is the multi-armed bandit problem (MAB), which has been widely explored and extended across multiple domains (Sutton and Barto 1998; Liu 2024).

In behavioral experiments, humans demonstrate remarkable adaptability in such settings, integrating environmental uncertainty, generalizing across trials, and dynamically adjusting their learning rates. These behaviors reflect a diversity of cognitive strategies (Steyvers et al. 2009). Although Bayesian approaches often capture human behavior well Behrens et al. (2007); Schulz et al. (2020); Zhang and Yu (2013), they are challenging to map directly onto biologically realistic neural dynamics. Despite the existence of many algorithms with strong theoretical guarantees, most lack biological plausibility, particularly in their architectural assumptions and learning and choice mechanisms.

In this work, our aim was to design a minimal, biologically inspired architecture able to solve non-stationary MAB tasks. Specifically, we proposed a simple architecture composed of two interacting plastic populations of rate-based neurons producing choices through agreement, called the Neural Selection Agreement model (NSA). We evaluated it on four variants of the MAB problem, each differing in how reward probabilities evolved over time and in a wide range of arm counts, from a few options to over a thousand. For comparison, we also tested three standard algorithms.

Our results show the model’s ability to adapt to changing reward distributions and quickly recover performance over time. It reliably tracked reward-optimal options and sustained effective decision policies, matching the performance of established methods such as Thompson Sampling, $\epsilon$-Greedy, and Upper Confidence Bound (UCB). To better understand the behavior of the system, we analyzed its responses at varying levels of reward distribution entropy. In low-uncertainty settings, NSA quickly identified the rewarding option and adopted a greedy policy, similar to Thompson Sampling. In contrast, UCB maintained a higher degree of exploration. As uncertainty increased, the model exhibited a higher option entropy in its decisions, transitioning to a more exploratory strategy similar to UCB. Although this change modestly affected the NSA’s ability to switch arms in highly volatile environments, overall performance remained robust.

The strengths of our model can be traced in both the architecture and the learning paradigm, whose hyperparameters were optimized through an evolutionary process. Interestingly, the values found converged to solutions that can be mapped to plausible synaptic mechanisms. On the one hand, the neural dynamics, which rely on plastic connections and a consensus-like selection process. Particularly important was the choice of modulating the afferent connections to the value population *V* according to a nonlinear function dependent on the synaptic weight itself. In so doing, it was possible to implicitly evolve an effective option value policy for the trade-off between exploration and exploitation.

The neural response functions that emerged were characterized by a steep sigmoidal shape, which can be related to the saturation of the neural response once a certain threshold is crossed, a feature observed in biological network as class III neurons, in addition to being a common choice for artificial ones (Ratté et al. 2013; Ocker and Buice 2020; Apicella et al. 2021).

On the other hand, learning was structured as a nonassociative plasticity rule based on reward. Similarly to before, a non-linear function of synaptic weights played a critical role, specifically in defining the synapse-specific learning rate (Larsen and Jesper Sjöström 2015). Furthermore, the shape evolved of the learning rate function was inversely proportional to the synaptic weight, which can be related to the availability of resources in the synapse and its state, including size (Bartol et al. 2015; Ariel et al. 2012). An additional consideration is the inspiration from the functional role of the orbitofrontal cortex (OFC) and the anterior cingulate cortex (ACC), two important pre-frontal regions known to be involved in decision-making processes (Kennerley and Walton 2011; Khamassi et al. 2013).

In our work, we have explored ways in which an option value can be formed according to recent reward history and connection weights, updating the option representations. The OFC is known to represent different options, updating their values based on history and rewards (Luk and Wallis 2013; Kennerley and Walton 2011; Klein-Flügge et al. 2013).

Next, the NSA model relies on some inductive biases, such as the shape of the Gaussian-sigmoid function, that affect neural activity and the weight-dependent learning rate. These biases may be considered to implicitly encode a policy that dictates how the two populations interact, how new information should be incorporated into the update of the weight value, and what option to choose next. In fact, ACC has been associated with the evaluation of actions and the regulation of the balance between exploration and exploitation (Khamassi et al. 2013; Kolling et al. 2016).

Additionally, the generation of an option selection results from a temporal interaction of the activity of the two neural populations, before converging to a choices. In a similar direction, it has been observed that the OFC transiently visits chosen and unchosen options before committing (Rich and Wallis 2016). Furthermore, the dynamic interaction between the ACC and OFC has been linked to transient pre-stimulus activations, which bias decisions toward the most valuable option (Funahashi 2017; Marcos and Genovesio 2016; Balewski et al. 2023).

Despite promising results, there are some limitations to the model. First, we considered the high level of abstraction in the neuronal details, as we relied on simple point neurons with synapses modeled with relatively elementary functions, lacking the anatomical complexity of actual dendrites. In particular, NSA does not account for the presence of noise in neural dynamics, which is a well-known feature of biological neurons (Faisal 2017). Furthermore, the functional association with the pre-frontal cortical region is only moderate, although present. On the computational side, since our interest lay in the biological plausibility and evolution of adaptive meta-learning solutions, we used only a few well established and relatively simple algorithms as a reference and did not take into account more advanced variants such as VDBE Tokic (2010); Tokic and Palm (2011), *f-Discounted-Sliding-Window Thompson Sampling* (*f-dsw TS*) (Cavenaghi et al. 2021), and variants of $\epsilon$-Greedy (Qi et al. 2023).

Future work could involve comparison with more sophisticated algorithms, the introduction of a larger architecture, and more realistic neural dynamics, such as spiking neurons (Nunes et al. 2022).

## Figures and Tables

**Fig. 5:**
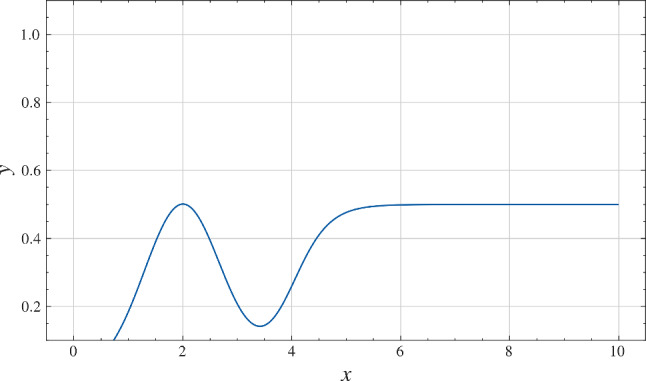
Activation function $\Phi _{v}$ - parameters $\beta =10$, $\alpha =1$, $\mu =1$, $\sigma =1$, and $r=0.5$.

**Algorithm 2:**
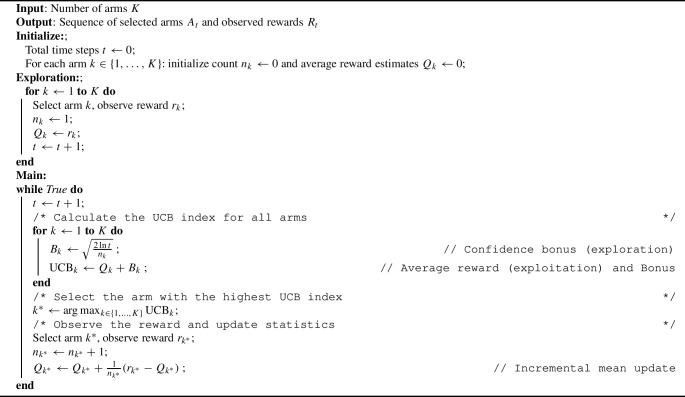
Upper confidence bound algorithm

**Algorithm 3:**
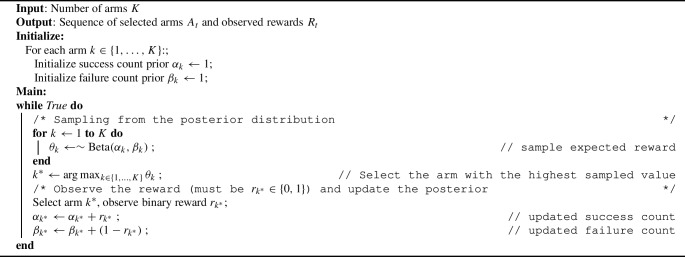
Thompson sampling algorithm

**Algorithm 4:**
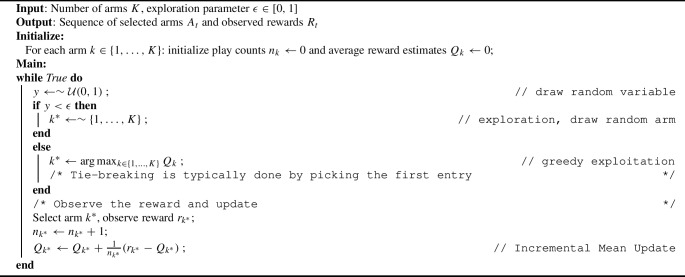
$$\epsilon $$-Greedy algorithm

**Fig. 6:**
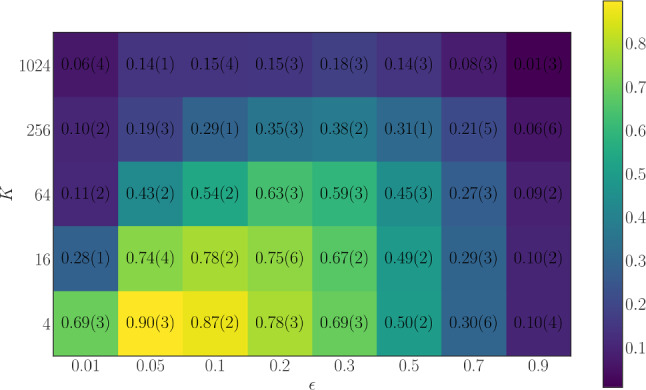
Sensitivity analysis of Epsilon-Greedy - performance of the algorithm on a piecewise stationary MAB for varying number of arms and $\epsilon$ parameter. In parentheses is reported the decimal position of the first significant digit

**Fig. 7:**
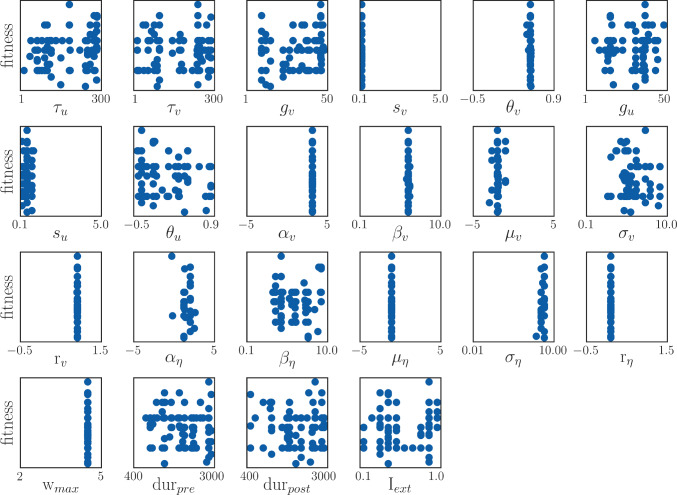
Genome distribution - each parameter is plotted against the fitness score

**Fig. 8:**
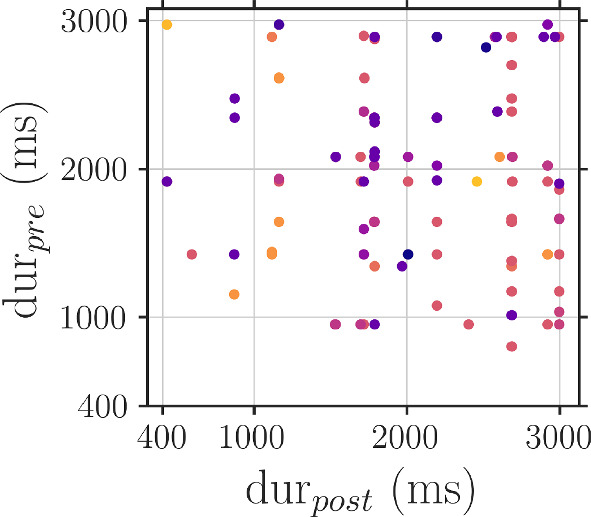
Phase durations and fitness - duration of the first and second phase, over the x and y axis respectively, for each individual of the top-scoring half of the population. The color indicates the fitness, with yellow being the highest.

**Fig. 9:**
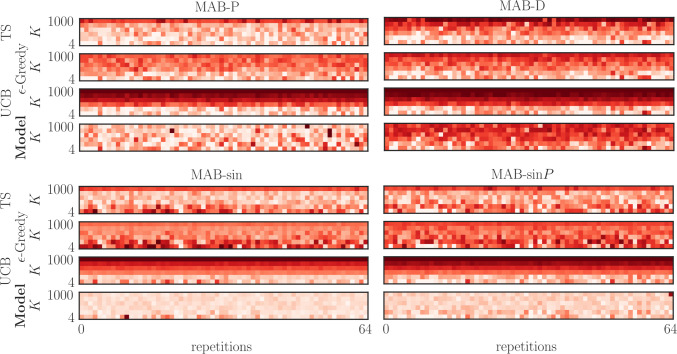
Heatmap of multiple runs - each single heatmap shows the average regret (white=low, dark red=high) of two trials for a single model on a given environment for a specific number of arms *K*

**Fig. 10:**
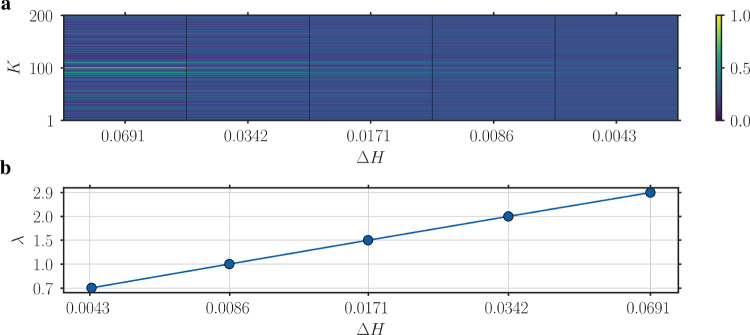
Showcase of distribution entropy - **a**: visualization of each distribution as heatmaps (columns), the entropy differences (x-axis) and arm indexes (y-axis); note that the entropy of distribution *i* is inversely proportional to the difference $\Delta H$ - **b**: log-log plot of the entropy differences (x-axis) against the scaling $\lambda$ of the top-arm with respect to the others (y-axis)

**Algorithm 5:**
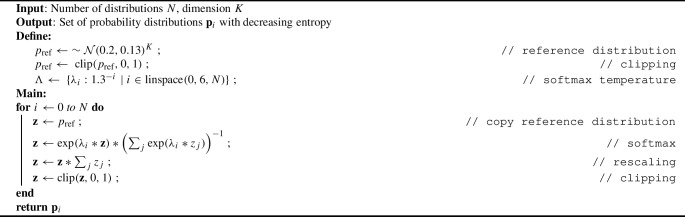
Reward probability distribution generation

**Fig. 11:**
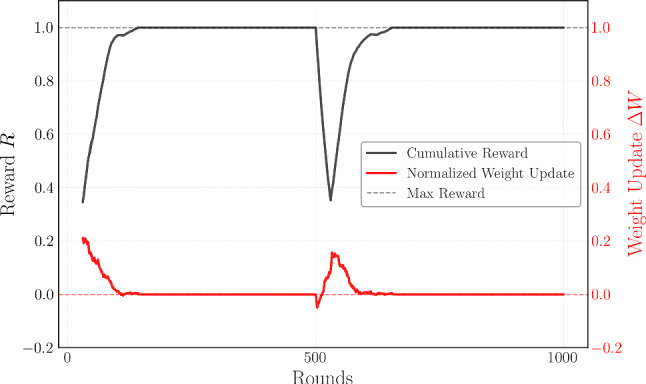
Weight update development for the model. The plot displays the weight update quantity $\Delta W_{k}^{UV}$ for each round (blue line), smoothed as a 20-step moving average. It is also reported the average reward in a window of 30 rounds (orange line). The results have been obtained averaging over 30 iterations

## Data Availability

No datasets were generated or analysed during the current study.

## Appendix

### Neural response function

The activation functions applied to the two neuronal populations are defined as a step-function composed with a generalized sigmoid as follows:

4 $$\begin{aligned} f(x; g, o, \theta ) = {\left\{ \begin{array}{ll} \left[ 1 + e^{-g(x - o)}\right] ^{-1} & \text { if } \left[ 1 + e^{-g(x - o)}\right] ^{-1} > \theta \\ 0 & \text { otherwise} \end{array}\right. } \end{aligned}$$

Where:

- *x* is the neuron pre-activation value
- *g* is the gain
- *o* is the offset
- $\theta$ is the threshold

Each population has its own set of parameters, which are optimized through evolutionary search.

### Gaussian-sigmoid function

The function $\Phi _{\cdot }$ is defined by combining a generalized version of the sigmoid, namely with a gain $\beta \ne 1$ and offset $\alpha \ne 0$, and a Gaussian with mean $\mu$ and variance $\sigma ^{2}$. Their contributions are weighted by *r* and $1-r$ ($r\in (0,1)$) respectively.

$$\begin{aligned} \Phi _v(x) = r\left( 1 + \exp ^{-\beta (x-\alpha )}\right) ^{-1} + (1-r)\exp \left( -\frac{(x-\mu )^2}{2\sigma ^2}\right) \end{aligned}$$

The motivation behind this choice is to express a function that possesses a bounded region (depending on $\mu ,\,\sigma$) at a high/low peak (depending on the value of $\gamma _{2}$), and a continuous transition to a constant value (depending on the steepness of the sigmoid $\beta$, shift $\alpha$, and intensity $\gamma _{1}$) (Fig. 5).

### Baseline literature models

The algorithms we chose to compare our model with are well-known in the literature of machine learning and reinforcement learning, although not representing the current state-of-the-art. They were implemented in native Python, and only $\epsilon -$Greedy required hyper-parameter calibration.

#### Epsilon-Greedy ($\epsilon$-Greedy)

Since this algorithm relies on a specific choice of the exploration hyper-parameter $\epsilon$, we conducted a grid search over a sample of arm numbers *K* for a range of $\epsilon$ values. Figure 6 shows the results of this search as a heatmap, which suggests a positive direct relationship between the two variables. Subsequently, we modified the $\epsilon -$Greedy implementation so that $\epsilon$ is set according to the number of arms, using the function $0.4*\log _{2}(0.6*K)$, whose form and parametrization have been fit from the search data.

### Evolutionary search

The optimization was carried out over several parameters concerning the model architecture, its dynamics, as well as three hyper-parmeters of the selection process. The following is the entire set of evolved values, with in parenthesis the range in which they were allowed to be sampled from. The specific choice of range has been chosen by trial and error, so that the ending distribution would not cluster over the positive or negative (if allowed) boundary, as shown in 7.

**Network parameters**

- $\tau _{u}\in (1, 300)$: time constant of population *U*
- $\tau _{v}\in (1, 300)$: time constant of population *V*
- $g_{u}\in (1, 50)$: gain of the neural response function of population *U*
- $g_{v}\in (1, 50)$: gain of the neural response function of population *V*
- $s_{u}\in (0.1, 5)$: offset of the neural response function of population *U*
- $s_{v}\in (0.1, 5)$: offset of the neural response function of population *V*
- $\theta _{u}\in (-0.5, 0.9)$: threshold of the neural response function of population *U*
- $\theta _{v}\in (-0.5, 0.9)$: threshold of the neural response function of population *V*
- $W^{+}\in (2, 5)$: maximum weight value for the weights ${\textbf {W}}^{UV}$

**Option value function parameters**

- $\beta _{v}\in (0.1, 10)$: steepness of the sigmoid
- $\alpha _{v}\in (-5, 5)$: shift of the sigmoid
- $\mu _{v}\in (-5, 5)$: mean of the Gaussian
- $\sigma _{v}\in (0.01, 10)$: variance of the Gaussian
- $r_{v}\in (-0.5, 1.5)$: weight of the sigmoid

**Learning rate function parameters**

- $\beta _{\eta }\in (0.1, 10)$: steepness of the sigmoid
- $\alpha _{\eta }\in (-5, 5)$: shift of the sigmoid
- $\mu _{\eta }\in (-5, 5)$: mean of the Gaussian
- $\sigma _{\eta }\in (0.1, 10)$: variance of the Gaussian
- $r_{\eta }\in (-0.5, 1.5)$: weight of the sigmoid

**Option selection process hyper-parameters**

- $\text {dur}_{\text {pre}}\in (400, 3000)$: duration of the first phase
- $\text {dur}_{\text {post}}\in (400, 3000)$: duration of the second phase
- $I_{\text {ext}}\in (0.1, 1)$: magnitude of the external input

Each individual has been evaluated over two environments using the following settings:

- MAB-0: average reward distribution entropy $\langle H\rangle =2.05$
- KAB-$\sin$ P: average reward distribution entropy $\langle H\rangle =2.1$, given *K* arm frequencies $f_{k}$ as an equally spaced set $\{0.1\ldots i\ldots 0.4\}$, phases $\lambda _{k}$ drawn from an uniform $\sim \mathcal {U}(0, 2\pi )$, and half of the arms have been set to constant values drawn from another uniform $\sim \mathcal {U}(0.1, 0.7)$; the final reward distribution was not normalized.

The number of arms was $K=40$ and 200, and lasted for 2 trials with 2000 rounds each. The final fit was the average of the 2 iterations.

The optimization has been implemented in Python using the DEAP library and the algorithm used was the CMA-ES algorithm. Optimization involved 40 generations with a population size of 256 individuals. The mutation rate was set at 0.5 with a sigma of 0.8, the cross-over rate was set at 0.4. The runs were carried out on a 256-core AMD EPYC 7763 with 2TB of RAM.

#### Genome distribution

Following the evolutionary search, the distribution of parameters over the top-scoring half of the population is taken, corresponding to 128 individuals.

Fig. 7 reveals the parameters shared among the models with the highest fitness score and those that are more variable. The most stable parameters are, predictably, those involved in directly shaping the neural activity (neural activation functions) and learning policy (Gaussia-sigmoid).

For what concerns the option selection algorithm 2.2.1, the values for the duration of the two phases were of modest importance, with the distribution being weakly skewed toward longer times. As shown in 8, fitness is also apparently not associated with particular values for either phase.

The effect of different durations for the first and second phases on performance is limited, as shown in Fig. 8.

### Multiple runs on multiple environments

The results reported in the table of results 1 were obtained by running all models in all environments on a variable number of arms multiple times (64). Figure 9 illustrates the results of the tests for each simulation setting.

In particular, the NSA model performs the worst in the piecewise stationary with drift scenario (MAB-D), while outcompeting the others in the sinusoidal cases and when *K* is large.

### Reward distribution entropy

The calculation of a set of *N* reward probability distribution $\textbf{p}_{i}\text { for } i\ldots N$ for *K* values with a progressive level of entropy $\textbf{h}_{i}\text { for } i\ldots N$ has been obtained by modifying a starting reference distribution $p_{\text {ref}}$. A description of the algorithm is presented below 5.6.

Given the reference $p_{\text {ref}}$ and its entropy $H_{\text {ref}}$, it is possible to quantify the difference in entropy with respect to each derived distribution *i* as $\Delta H=H_{\text {ref}} - H_{i}$. This measure is informative about how much the best arm stands out from the others.

In Fig. 10 are illustrated examples of distributions with decreasing levels of entropy difference with the reference by means of a heatmap in 10a. Both plots highlight the blending of the probability associated with the best arm with the other ones, eventually becoming indistinguishable from the rest. In the MAB tasks, this increase in entropy translates into a higher difficulty in picking the best option, although the other side of the coin is that regret also decreases, since the cost of selecting a non-optimal arm is less impactful on performance. Plot 10b shows the relationship between the scaling of the difference between the best arm and the rest.

### Weight update dynamics

We also analyzed the weight update dynamics of the model over the rounds. In Fig. 11, we plotted the evolution of the total weight $\Delta W^{UV}$ over time, averaged over 20 simulations, and smoothed over 30 rounds. The results show that the model can quickly adapt to new reward distributions. It is also able to maintain the optimal policy over time, with the weights remaining approximately stable. The quantity of updates $\Delta W_{k}^{UV}$, which in each round is applied to one connection *k*, changes sign according to the collected reward, with its magnitude higher at the beginning of the trials. Initially, the sign is mostly positive (potentiation) since the weights start at zero, and after some uncertainty a consistently preferred arm emerges. However, when the reward distribution switches, a regular series of suboptimal choices is made with respect to the new distribution, leading to zero reward. This causes an accumulation of negative sign weight updates (depression), eventually causing the value of the preferred arm to drop. In the meantime, other options are probed until another sequence of choices converges to another arm, promoted by a trail of positive weight updates.

This behavior is consistent with the low entropy levels observed in the previous analysis.

## Footnotes

1 Since the arm probabilities are not normalized to 1, it is technically improper to call them *probability distributions*; we therefore refer to either *probabilities* or *distributions*, depending on the context, to avoid confusion.
